# Animal-assisted intervention in the ICU: a tool for humanization

**DOI:** 10.1186/s13054-018-1946-8

**Published:** 2018-02-12

**Authors:** Megan M. Hosey, Janice Jaskulski, Stephen T. Wegener, Linda L. Chlan, Dale M. Needham

**Affiliations:** 1 Department of Physical Medicine and Rehabilitation, Division of Rehabilitation Psychology and Neuropsychology, Johns Hopkins School of Medicine, Baltimore, MD USA; 2 Outcomes After Critical Illness and Surgery (OACIS) Group, Johns Hopkins School of Medicine, Baltimore, MD USA; 3 Mayo Clinic College of Medicine and Science, Mayo Clinic, Rochester, MN USA; 4 Division of Pulmonary & Critical Care Medicine, Department of Physical & Rehabilitative Medicine, Johns Hopkins School of Medicine, Baltimore, MD USA; 5 Critical Care Physical Medicine & Rehabilitation Program, Johns Hopkins School of Medicine, Baltimore, MD USA

The combination of an aging population and advances in critical care medicine is resulting in a growing number of survivors of critical illness [1]. Survivors’ descriptions of their stay in an intensive care unit (ICU) are frequently filled with traumatic events, and include experiences of confusion, anxiety, sleeplessness, pain, and loneliness [2, 3]. Sedative and anxiolytic medications administered to manage patient symptoms are associated with delirium and worse physical and mental health outcomes [4]. Therefore, there is growing interest in the use of non-pharmacologic interventions and in creating a more humanized environment in the ICU for patients and their families [5]. Such efforts have included a focus on understanding the critically ill patient as an individual and providing comprehensive medical, psychological, and rehabilitation care [6–8]. This publication aims to: 1) suggest a conceptual model for the use of non-pharmacologic interventions to reduce suffering and promote recovery in a more humanized ICU environment; 2) describe animal-assisted intervention (AAI) as an exemplar of a non-pharmacologic intervention and provide a conceptual model for the utility of this intervention; and 3) discuss the basic principles for introducing a non-pharmacologic intervention program in the ICU.

## Patient suffering and the humanized ICU: where do non-pharmacological interventions fit?

**Fig. 1:**
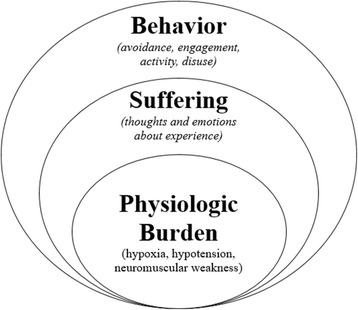
Application of non-pharmacological intervention in the humanized ICU may lead to reduced physiologic burden, less suffering, and more engaged behavior with reciprocal effects in each domain

To aid in conceptualizing non-pharmacologic interventions in the ICU, we propose an adaptation of the Loeser pain and suffering model [9]. This model highlights the inter-relatedness of physiologic and emotional suffering, and the importance of interdisciplinary care in recovery from disease (Fig. 1). In the model, the innermost circle represents *physiologic burden* where patients sustain physiologic changes, such as hypoxia or hypotension, and require medical interventions, such as mechanical ventilation or vasopressors. The second circle represents *suffering*, which includes the patient’s thoughts (e.g., “I am short of breath. I am dying;” “I am a burden and worthless;” “Walking while critically ill will harm me”) and emotions (e.g., anxiety, sadness, and loneliness) about their physiologic and environmental experience. Non-pharmacologic interventions to alleviate suffering can include education, psychological support, and other methods to reduce distress (e.g., cognitive-behavioral therapy, animal-assisted intervention, and music intervention). The third circle represents *behavior*, wherein worsening of physiologic burden and suffering can change patient engagement in medical and/or rehabilitation care (e.g., disengagement in rehabilitation, avoidance of medical information, declining recommended medical interventions). In the behavior realm, interventions (e.g., early mobility and motivational interviewing) move patients toward action and reinforce their role as participants in their own recovery.

Interventions in one circle have the potential to influence outcomes in other domains. Equally important is acknowledging that the patients’ experiences at each level are *real* even if they are difficult to observe and measure. Increased attention to both patient suffering and behavior domains ensures comprehensive care and potentially better long-term outcomes.

## AAI: an exemplar of a non-pharmacologic intervention to reduce suffering and encourage recovery behavior

**Fig. 2:**
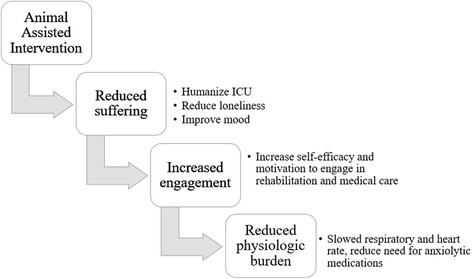
Animal assisted intervention as an example of non-pharmacologic intervention to reduce suffering with potential downstream benefits

**Fig. 3:**
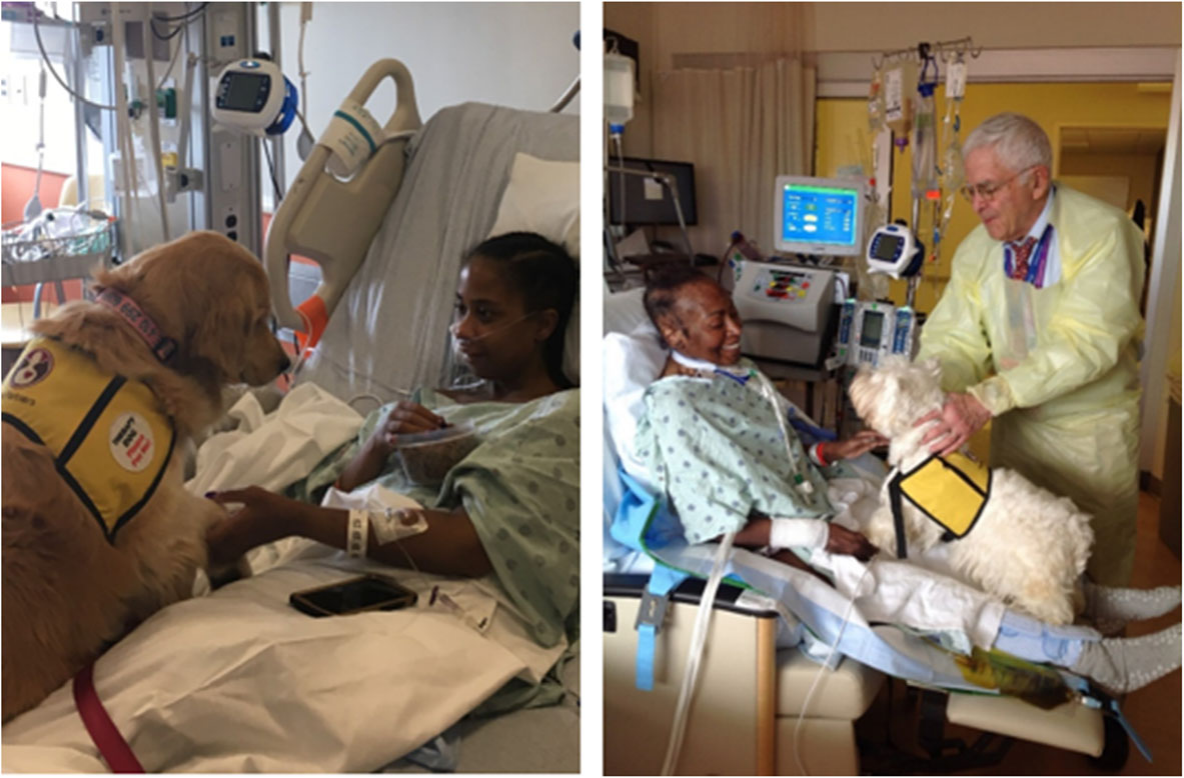
Patient with pain after chest tube removal gets distraction and relaxation with Winnie, the Golden Retriever (*left*), and patient receiving continuous renal replacement therapy and mechanical ventilation via tracheostomy finds motivation to sit out of bed in a chair thanks to Pippi, the West Highland Terrier (*right*)

Some healthcare facilities have integrated AAI, in populations ranging from pediatrics to geriatrics, in order to reduce suffering and promote recovery behavior. Existing literature suggests that AAI reduces symptoms of anxiety and depression [10, 11], promotes engagement in rehabilitation therapies [12], and eases distressing physiologic symptoms (e.g., pain) [13]. Data regarding AAI in the ICU are scant, with narratives suggesting that animal presence is beneficial to patients [14]. Hypothesized mechanisms for the benefit of AAI (and potentially other non-pharmacologic interventions) are outlined in Fig. 2. Further research regarding potential benefits is needed to build the case for animal presence in the humanized ICU. Anecdotal evidence suggests that a dog sitting in a patient’s lap eases suffering and builds motivation in ways that medical interventions may not (Fig. 3).

## Implementing an AAI program for the ICU

Building new, non-pharmacologic interventions, with the intention to reduce suffering and optimize health behavior change, takes a concerted, multidisciplinary effort. Although we use the exemplar of AAI, the following program building process may apply to other non-pharmacological interventions.

We have identified six critical success factors for program building: (1) designating a champion who is consistently present in the ICU with established credibility to create systematic change; (2) having clear program goals with milestones and measurable outcomes, such as (a) improving patient mood, (b) improving engagement in medical care and rehabilitation therapies, and (c) reducing perceived pain; (3) including stakeholders who can help identify and surmount barriers to implementation (Table 1), such as risk management and hospital epidemiology and infection control staff; (4) identifying animal teams and partnering them with an organization that has credibility in training teams for the hospital environment, such as Pet Partners, Inc. (https://petpartners.org/) and Assistance Dogs International (https://assistancedogsinternational.org); (5) creating a policy that (a) establishes goals of the program, (b) outlines roles/responsibilities for all involved in the program, (c) outlines logistics of animal visits, (d) specifies what do in the event of an accident, and (e) establishes a plan for program evaluation; and (6) launching the program with patients who have a high likelihood of success, such as patients without delirium, communicable disease, or the need for contact precautions due to colonization with a drug-resistant microorganism, to build confidence and create momentum for the program.

**Table 1:** Examples of stakeholders and roles for an AAI program

Stakeholder	Role and responsibilities
Program Champion	1. Develops policy and procedures with the healthcare facility stakeholders
	2. Provides training for facilitators of AAI interventions and ensures that protocol is adopted/followed
	3. Coordinates and/or oversees visits
	4. Oversees program evaluation
ICU team	1. Ensures patient/family appropriateness for visit. Recommended questions when evaluating patient for AAI:
	a. Is the patient interested?
	b. Is the patient able to benefit (e.g., assess cognitive status)?
	c. Is the patient on infection-related contact precautions?
	2. Places consult request for AAI
	3. Coordinates timing of AAI to fit patient schedule and ICU workflow
Risk management	1. Ensures patient privacy (HIPAA)
	2. Provides guidance about prevention/management of patient injury; recommendations include:
	a. Using certified therapy animal teams
	b. Limiting length/number of patient visits per animal visit
	c. Ensuring liability insurance in place
	3. Provides guidance about prevention/management of animal injury or death
Infection control	1. Protects patients from zoonotic infection; [15] recommendations include:
	a. Mandating annual veterinary examination, fecal test for infection and parasites, up-to-date vaccinations
	b. Bathing/grooming the animal before and after each hospital visit
	c. Prohibiting animals with any illness within 24 h of visit
	d. Prohibiting animals with an open wound
	2. Protecting from fomite infection; recommendations include:
	a. Washing hands for patients, staff members and visitors before and after touching animal
	b. Cleaning animal toys after use
	3. Excluding or using special precautions for specific patient groups, including those:
	a. Known to be colonized or infected with multi-drug resistance bacteria (e.g., methicillin-resistant *Staphylococcus aureus*), *Clostridium difficile*, or tuberculosis
	i. Special precaution: animal only visits one patient or visits the infected/colonized patient last (with approval from infection control)
	b. Who have open wounds or a wound vacuum
	i. Special precaution: cover wound; avoid animal having contact with wound
	c. Who are immunocompromised

## Summary

As critical care medicine is increasingly successful in preventing death, the field is more focused on optimizing patients’ survivorship experience. Through creating humanized ICU environments and implementing non-pharmacologic interventions, patients no longer must wait for hospital discharge before they begin to live again. Non-pharmacological intervention programs, such as AAI, may reduce suffering and help patients take an active role in their recovery.

## References

[1] Adhikari NK, Fowler RA, Bhagwanjee S, Rubenfeld GD (2010). Critical care and the global burden of critical illness in adults. Lancet..

[2] Eakin MN, Patel Y, Mendez-Tellez P, Dinglas VD, Needham DM, Turnbull AE (2017). Patients’ outcomes after acute respiratory failure: a qualitative study with the PROMIS framework. Am J Crit Care.

[3] Chahraoui K, Laurent A, Bioy A, Quenot J-P (2015). Psychological experience of patients 3 months after a stay in the intensive care unit: a descriptive and qualitative study. J Crit Care..

[4] Reade MC, Finfer S (2014). Sedation and delirium in the intensive care unit. N Engl J Med..

[5] Brown S, Beesley S, Hopkins R. Humanizing intensive care: theory, evidence, and possibilities. In: Jean-Louis Vincent, editor. Annual update in intensive care and emergency medicine 2016. Switzerland: Springer International Publishing; 2016. p. 405–20. 10.1007/978-3-319-27349-5.

[6] Chlan L (2002). Integrating nonpharmacological, adjunctive interventions into critical care practice: a means to humanize care?. Am J Crit Care..

[7] Haines KJ, Kelly P, Fitzgerald P, Skinner EH, Iwashyna TJ (2017). The untapped potential of patient and family engagement in the organization of critical care. Crit Care Med..

[8] Jackson JC, Santoro MJ, Ely TM, Boehm L, Kiehl AL, Anderson LS (2014). Improving patient care through the prism of psychology: Application of Maslow’s hierarchy to sedation, delirium, and early mobility in the intensive care unit. J Crit Care..

[9] Loeser JD (2000). Pain and suffering. Clin J Pain..

[10] Bernabei V, De Ronchi D, La Ferla T, Moretti F, Tonelli L, Ferrari B (2013). Animal-assisted interventions for elderly patients affected by dementia or psychiatric disorders: a review. J Psychiatr Res..

[11] Hoffmann AO, Lee AH, Wertenauer F, Ricken R, Jansen JJ, Gallinat J (2009). Dog-assisted intervention significantly reduces anxiety in hospitalized patients with major depression. Eur J Integr Med..

[12] Lasa SM, Ferriero G, Brigatti E, Valero R, Franchignoni F (2011). Animal-assisted interventions in internal and rehabilitation medicine: a review of the recent literature. Panminerva Med..

[13] Halm MA (2008). The healing power of the human-animal connection. Am J Crit Care Off Publ Am Assoc Crit-Care Nurses..

[14] Lee D, Higgins PA (2010). Adjunctive therapies for the chronically critically ill. AACN Adv Crit Care..

[15] Lefebvre SL, Golab GC, Christensen E, Castrodale L, Aureden K, Bialachowski A (2008). Guidelines for animal-assisted interventions in health care facilities. Am J Infect Control..

